# Translation, Cross-Cultural Adaptation and Psychometric Validation of the Romanian Version of the Boston Carpal Tunnel Questionnaire

**DOI:** 10.3390/diagnostics15233036

**Published:** 2025-11-28

**Authors:** Nicu Catalin Draghici, Daniel-Corneliu Leucuța, Maria Gianina Balea, Roxana Toader, Livia Livint-Popa, Cristina Tecar, Diana Sipos-Lascu, Oliviu Florentiu Sarb, Tudor Dimitrie Lupescu, Dafin Fior Mureșanu

**Affiliations:** 1 Department of Clinical Neurosciences, “Iuliu Hatieganu” University of Medicine and Pharmacy, 400012 Cluj-Napoca, Romania; nicu.draghici@umfcluj.ro (N.C.D.); livia.popa@umfcluj.ro (L.L.-P.); dia_lascu@yahoo.com (D.S.-L.);; 2 IMOGEN Institute, Centre of Advanced Research Studies, 400347 Cluj-Napoca, Romania; 3 RoNeuro Institute, Centre for Neurological Research and Diagnostic, 400364 Cluj-Napoca, Romania; 4 Department of Medical Informatics and Biostatistics, “Iuliu Hatieganu” University of Medicine and Pharmacy, 400349 Cluj-Napoca, Romania; 5 Department of Epidemiology, “Iuliu Hatieganu” University of Medicine and Pharmacy, 400348 Cluj-Napoca, Romania; sarboliviu@yahoo.com

**Keywords:** carpal tunnel syndrome, Boston Carpal Tunnel Questionnaire, Romanian version of the BCTQ, validity

## Abstract

**Background/Objectives**: Carpal tunnel syndrome (CTS) is the most common peripheral neuropathy, often impairing daily function and quality of life. This study aimed to translate, cross-culturally adapt, and assess the validity of the Boston Carpal Tunnel Questionnaire (BCTQ) in a Romanian cohort. **Methods**: We conducted a cross-sectional study to translate, culturally adapt, and validate the Romanian version of the BCTQ. A total of 190 participants were recruited, representing 380 wrists, including 130 patients with suspected or confirmed CTS and 60 healthy controls. The adaptation process followed international standards, with forward and backward translation, expert committee review, and pilot testing. Participants completed the Romanian BCTQ and performed standardized nerve conduction studies. The internal consistency and validity, including convergent and discriminant validity, exploratory factor analysis, and test–retest assessments, were performed. **Results**: The Romanian BCTQ exhibited exceptional internal consistency (Cronbach’s α = 0.96), significant inter-item correlations, and robust factor loadings confirming a one-factor structure. Convergent validity was evidenced by strong correlations with electrodiagnostic measurements. The test–retest reliability was high, with intraclass correlation coefficients above 0,99. **Conclusions**: The Romanian adaptation of the BCTQ is a valid, reliable, and culturally appropriate patient-reported instrument to assess symptom severity and functional status in CTS, improving clinical decision-making and cross-cultural research.

## 1. Introduction

Carpal Tunnel Syndrome (CTS) is the most prevalent peripheral nerve disease in the upper extremities [1,2]. It involves the compression of the median nerve at the wrist, as it passes through the carpal tunnel. This injury results in impaired function, numbness, and paresthesia in the hand, especially in the thumb, index finger, and middle finger, as well as positive Tinel’s and Phalen’s tests [3].

CTS has emerged as a significant public health concern in Romania due to long-term rehabilitation, diagnostic procedures, and increasing surgical demand. The CTS disproportionately impacts women living in rural areas, factory workers, and professionals working over 12 h daily at the computer [4,5,6]. Along with that, repetitive strain and occupational hand overuse have been identified as key factors in the development of carpal tunnel syndrome [7]. In addition, rheumatoid arthritis, diabetes, and metabolic syndrome significantly contribute to increased susceptibility to comorbidity-driven neuromuscular disorders, particularly among Romanian women [4,8,9,10]. Moreover, studies show that women are more at risk than men of suffering from carpal tunnel syndrome, with a 3:1 female predominance in both clinical presentations and surgical cases. Also, the incidence is higher in women aged 40–60, with the average age for surgery being mid-50s [4,10]. The condition burdens most women living in rural areas, highlighting challenges in female-dominated manual labor industries where early intervention is often inaccessible or unaffordable [4,5]. Although improvements are being made with validated Romanian versions of symptom scales supporting standardized CTS screening, these diagnostic tools remain underutilized in primary care, resulting in frequent oversight of early symptoms across a range of neuropathies [8,11,12].

The diagnosis of CTS is based on history, clinical signs, and symptoms [13,14]. This should then be followed by electrodiagnostic (EDX) testing (such as electroneuromyography) or imaging (such as magnetic resonance imaging or ultrasound) [2].

This study aimed to translate, cross-culturally adapt, and assess the validity of BCTQ in a Romanian cohort.

## 2. Materials and Methods

### 2.1. Study Design and Setting

The present study was a cross-sectional, observational study aimed at developing and culturally adapting a Romanian version of the Boston Carpal Tunnel Questionnaire (BCTQ) and validating its clinical utility through psychometric testing against established diagnostic and functional assessment standards.

The study was conducted at the RoNeuro Institute for Neurological Research and Diagnostic and the IMOGEN Center of Advanced Research Studies in Cluj-Napoca, Romania, between October 2023 and November 2024. A previous study on a 31-participant sample attempted a Romanian adaptation of the BCTQ [15]. Nevertheless, to achieve full psychometric validation of the BCTQ, our study included a total of 190 participants to enable factor-analytic stability (~10 participants per item), precise reliability estimates (internal consistency and test–retest), and robust known-groups validity. Our study design aligns with international gold standards for questionnaire validation, which emphasize the necessity of large, representative samples (≥100–200 participants, with controls) to enable comprehensive psychometric evaluation, including internal consistency, responsiveness, and structural validity [16,17,18,19].

### 2.2. Ethical Approval

The study was approved by the Ethics Committee of the” Iuliu Hațieganu” University of Medicine and Pharmacy (Cluj-Napoca, Romania), reference number 20/17.02.2024, approval date 19 February 2024.

### 2.3. Translation and Cultural Adaptation

Since its development by Levine et al. in 1993, the BCTQ has been translated and validated in multiple languages and cultural contexts, reflecting its global acceptance as a patient-reported outcome measure (PROMS) for CTS [20].

In this study, the Romanian adaptation of the BCTQ followed the Beaton/AAOS framework, an internationally recognized methodology considered the gold standard in health outcomes research [17]. This structured approach emphasizes methodological rigor to achieve semantic, conceptual, and cultural equivalence, thereby ensuring that the adapted tool is both scientifically robust and clinically meaningful [21]. The adaptation process applied to the Romanian version was carried out in the following stages: forward translation, review and synthesis, linguistic quality check, back translation, expert committee review and validation, and final validation and reliability assurance. Please consult Supplementary Table S1 for more details. The translation can be found in Appendix A.1. and Appendix A.2.

### 2.4. Study Participants

**Figure 1:**
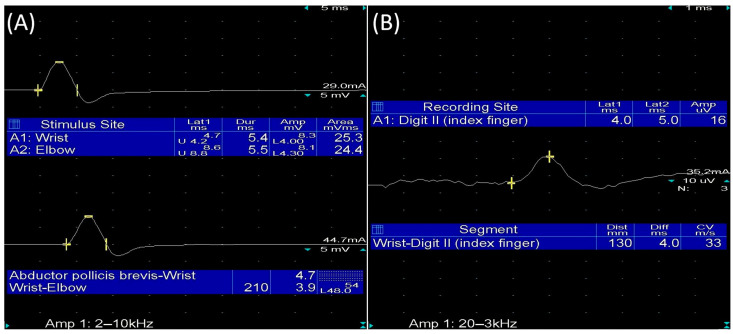
Median motor and sensory nerve conduction: (**A**) Median motor nerve conduction: Motor response recorded from the abductor pollicis brevis muscle after wrist stimulation (distal latency > 4.2 ms). (**B**) Median sensory nerve conduction: Sensory response recorded from digit II after wrist stimulation (velocity < 40 m/s).

The initial protocol aimed to enroll Romanian-speaking participants, with intermittent or constant numbness and/or tingling in at least 2 of digits 1, 2, 3, and 4 for at least 1 month [22]. At the same time, the best diagnostic strategy for CTS remains uncertain; consequently, to increase the diagnosis accuracy and to exclude other causes of neuropathy, we used EDX evaluation to confirm CTS [23]. Therefore, according to the criteria established by the American Association of Neuromuscular & Electrodiagnostic Medicine (AANEM), a distal motor latency > 4.2 ms in the median nerve and/or a sensory conduction velocity < 40 ms were considered pathological and diagnostic for CTS [22]. (Figure 1).

Patients were excluded if they did not speak Romanian, did not provide informed consent, or were unable to read or understand the study aims and procedures.

### 2.5. Data Collection

The following data points were recorded from each patient: age, gender, height, weight, residency (whether urban or rural), level of education, and dominant hand. Furthermore, patients were asked to declare when CTS symptoms first appeared, as well as whether they had any history of repetitive movement or working with vibrating tools. Afterward, subjects underwent a nerve conduction study (NCS), in which the following parameters were studied: median and ulnar Compound Muscle Action Potential (CMAP) amplitude in millivolts (mV). Median and ulnar CMAP latency in milliseconds (ms). For both nerves, the sensory function was assessed using the Sensory Nerve Action Potential (SNAP) amplitude in microvolts (µV), and antidromic median and ulnar nerve NCS velocity for Digit II and Digit V in meters per second (m/s). Lastly, all participants completed the Romanian version of BCTQ.

For the test–retest assessment, participants were asked to complete the questionnaire in about one to two weeks from the first evaluation.

### 2.6. Psychometric Evaluation

The psychometric evaluation of the Romanian BCTS involved the assessment of internal consistency and validity, including convergent and discriminant validity analyses.

### 2.7. Boston Carpal Tunnel Questionnaire (BCTQ)

BCTQ is a condition-specific, patient-reported outcome measure designed to assess symptom severity and functional status in patients with CTS. The questionnaire comprises two distinct subscales: the Symptom Severity Scale (SSS) and the Functional Status Scale (FSS). The SSS includes 11 items that evaluate various aspects of CTS symptoms, such as pain, numbness, tingling, and nocturnal awakenings due to discomfort. Each item is evaluated on a Likert scale ranging from 1 to 5 points, with higher scores indicating greater symptom severity. The FSS contains 8 items that measure the degree of difficulty in performing common daily activities, such as writing, buttoning clothes, and other daily activities, also scored on a 5-point Likert scale. Total scores are calculated by summing individual item scores and dividing this number by 11 and 8, respectively, within each subscale. The BCTQ has been widely used in clinical and research settings due to its strong psychometric properties. In the present study, the BCTQ was used as the primary instrument for assessing the severity of CTS symptoms and their impact on daily function in a Romanian-speaking population.

### 2.8. Nerve Conduction Studies

Three neurologists with expertise in neurophysiology conducted electrodiagnostic tests. Nerve stimulation was performed using a bar electrode, and responses were recorded using surface electrodes. Throughout the examination, skin temperature was maintained above 32 °C, and the skin was degreased beforehand to minimize potential grounding artifacts.

Thus, following the recommendations of E. Fournier, distal motor stimulation of the median nerve was performed at the wrist level, between the tendons of the palmaris longus and flexor carpi radialis muscles. The motor response was recorded from the abductor pollicis brevis muscle, localized on the lateral aspect of the thenar eminence. Moreover, standard motor conduction studies were conducted for the ulnar nerve. Distal stimulation was applied to the anterior part of the wrist, posterior to the flexor carpi ulnaris tendon, and recordings were collected from the abductor digiti minimi muscle at the hypothenar eminence level. Additionally, antidromic sensory nerve conduction velocities were calculated for both the median and ulnar nerves. Stimulation was performed at the radiocarpal joint at the aforementioned landmarks, with sensory recordings acquired from the second digit (for the median nerve) and the fifth digit (for the ulnar nerve) [24].

### 2.9. Statistical Analysis

Qualitative data were presented as absolute and relative frequencies. Normally distributed data were presented as means and standard deviations. Data not following the normal distribution were presented as medians and interquartile ranges. The priori sample size was calculated to ensure statistical power, and roughly 10 participants per question were aimed at in line with guidelines for standard validation studies. Having two wrists per participant meant there was excess sample above the minimum needed. For assessing internal consistency, Cronbach’s alpha and Guttman’s Lambda were calculated. Cronbach’s alpha, most commonly used, checks for consistency of items, while Guttman’s Lambda provides better estimation in conditions of item heterogeneity. According to Nunnally (1994), reliabilities of over 0.8 were considered acceptable [25]. The average inter-item correlation was also inspected, and a leave-one-out approach was followed to test whether removing each single item significantly impacted overall reliability. Exploratory factor analysis (EFA) was conducted using principal axis factoring and varimax rotation to derive the factor structure. Bartlett’s test of sphericity and Kaiser-Meyer-Olkin (KMO) measure of sampling adequacy were applied in assumptions checks.

For construct validity, convergent and discriminant validity were determined. Convergent validity was determined using Spearman correlations to compare the SSS and FSS to EDX severity measures (Median CMAP Latency and Antidromic Median NCS), since nerve conduction tests are the gold standard for measuring nerve dysfunction [26]. Divergent validity was determined using correlations with ulnar CMAP latency, which are not severity-related. The following criteria were applied to interpret correlations as weak (0–0.25), moderate (0.25–0.50), good (0.50–0.75), and excellent (0.75–1) [27]. In order to adjust for multiple tests, the Holm adjustment of *p*-values was applied. The test–retest assessment was conducted using the interclass correlation coefficient, using a two-way random effect model, for single unit observations, and measuring agreement, along with a 95% confidence interval and a *p*-value [28]. The comparison between the test and retest observations was performed with a Wilcoxon signed-rank test. Statistical analyses were conducted using version 4.3.1 of R (R Foundation for Statistical Computing, Vienna, Austria) [29], and psychometric tests were conducted using the psych package of R (version 2.5.3) [29], while the interclass correlation coefficient was computed with the irr package (version 0.84.1).

## 3. Results

A total of 190 participants were included in the study. The number of examined wrists was 380. Baseline demographic and clinical characteristics, including symptom duration and relevant occupational history, were recorded for all patients and are summarized in Table 1.

### 3.1. Exploratory Factor Analysis

The *p*-value for Bartlett’s test of sphericity was less than 0.001, indicating that there is sufficient correlation between the items to support the factor analysis for both SSS and FSS. The Kaiser-Meyer-Olkin (KMO) values of sampling adequacy were 0.92 for SSS and 0.93 for FSS, indicating that the dataset has a very strong factor structure, making it well-suited for exploratory factor analysis. Furthermore, the values for individual items are all above 0.87 for SSS and above 0.90 for FSS, indicating strong factorability for each variable.

All items load strongly onto the single factor, with values above 0.7, ranging from 0.73 to 0.88 for SSS and ranging from 0.84 to 0.91 for FSS (Table 2). The uniqueness values indicate that the factor accounts for the majority of the variance in each item, despite the fact that there is some variability across items. For SSS, the ninth and the first item had the lowest uniqueness, indicating that these factors account for the most of its variation, while the final (11) item and the fifth one had the greatest uniqueness, with the common factor explaining the least amount of its variance. For FSS, the sixth item had the lowest uniqueness, indicating that this factor accounts for the most of its variation, while the first item had the greatest uniqueness, with the common factor explaining the least amount of its variance. All things considered, the common underlying concept is well captured by the one-component model for both SSS and FSS.

The mean symptoms range from 1.5 to 2.1, and the standard deviations are around 1 for SSS (Table 2). For FSS, the means are lower, ranging from 1.3 to 1.7, and smaller standard deviations, around 0.8. Each item correlates with the total scale, indicating strong contributions to the scale, with values above 0.7 for both scales.

### 3.2. Reliability

#### 3.2.1. Item Statistics

The reliability of BCTQ SSS and FSS was excellent, with a Cronbach’s alpha of 0.96 (95% CI 0.95–0.96) and 0.96 (95% CI 0.95–0.96), respectively. Gutmann’s Lambda offered a similar result concerning reliability, with a value of 0.97 for SSS and 0.96 for FSS. The strong internal consistency is further supported by the average inter-item correlation of 0.67 for SSS and 0.76 for FSS. The Cronbach’s alpha remains around 0.95–0.96 even if any item is removed for both scales, suggesting that no single item significantly weakens the scale.

#### 3.2.2. Validity

Convergent validity was demonstrated in the form of statistically significant adjusted correlations to electro-physiological tests, as indicated in Table 3. These findings add strength to concurrent criterion validity. Specifically, there was a moderate inverse proportional correlation of both SSS and FSS and the Antidromic Median NCS of digit II. This indicates that higher SSS and FSS levels yielded lower Antidromic Median NCS levels. Conversely, there was a moderate direct proportional association of SSS and FSS to the Median CMAP Latency, which was reflective of higher latencies of the median nerve in conjunction with higher symptom scores. It is important to note that the correlation coefficients of FSS were marginally smaller than in the case of SSS, which reflected a relatively less tight association. In turn, divergent validity was indicated in the absence of significant adjusted correlations of measured symptom scores to Ulnar CMAP Latency, which reflected that the studied constructs differed and failed to present significant overlap with extraneous EDX variables.

#### 3.2.3. Test–Retest Reliability

The test–retest reliability was measured using the interclass correlation coefficient, having excellent values, above 0.80, for both SSS and FSS (Table 4).

## 4. Discussion

The current research sought to translate, culturally adapt, and validate BCTQ to apply to Romanian-speaking participants. The results confirm that the Romanian version has sound psychometric properties, which reflect high validity and reliability. The questionnaire had high internal consistency, with high intercorrelations of single items and total score, and high factor loadings, which confirm the consistency of the one-factor structure of the original CTS symptoms and function construction. The research also demonstrated convergent and concurrent criterion validity, in that the measure was significantly correlated with EDX measures. The divergent validity was also demonstrated in relation to EDX measures, further indicating the efficiency of the tool in differentiating concepts that are related and not. The tool also demonstrated high test–retest measurement agreement.

Previous cultural adaptations (*n* = 22) have been compiled in Supplementary Table S2.

BCTQ translation and validation across cultures support its acceptance as a patient-reported outcome measure in CTS. The variety of adaptations includes Arabic [21,30], Bulgarian [31], Chinese [32], Dutch [33], Finnish [34,35], Greek [36], Brazilian Portuguese [37,38], Polish [39], Korean [40,41], Serbian [42], Slovakian [43], Spanish [44] and Turkish [45,46]. In addition, Australian clinical adaptations of the Functional Status Scale and Symptom Severity Scale have been produced for routine use (Metro North Hospital and Health Service, 2016a, 2016b) [47,48]. These instruments were developed by Metro North Hospital and Health Service in 2016 as internal standardized tools for functional and symptom monitoring in Brisbane hospitals. They have not been published or independently validated, and no psychometric testing has been reported.

Consolidated, these studies demonstrate that the BCTQ maintains good reliability, validity, and responsiveness across diverse cultural settings, though most validations relied on modest sample sizes, often recruited from single clinical sites, which may limit generalizability. Furthermore, in the context of the other results and compared to the Serbian translation, the Romanian version achieved slightly higher internal consistency (Cronbach’s α of 0.96 and 0.96 compared to 0.91 and 0.93 for SSS and FSS, respectively), as well as test–retest reliability (ICC of 0.993 and 0.998 compared to 0.949 and 0.959 for SSS and FSS, respectively) [42]. This was also true when comparing the current version with the Thai version (Cronbach’s α of 0.91 and 0.94, respectively, and ICC of 0.89 and 0.98, respectively) [49]. Lastly, the Romanian version of BCTQ achieved higher internal consistency than its Polish counterpart (which had Cronbach’s α of 0.91 and 0.93 for SSS and FSS, respectively), as well as higher test–retest reliability (the Polish version having ICC values of 0.69 and 0.55 for SSS and FSS, respectively) [50] Of note, the Polish version of BCTQ was then used to validate the Hand Function Scoring system used in hand injury treatment and rehabilitation [39].

During the research process, we encountered only minor problems in translation and cultural adaptation. The questionnaire contains items that describe symptom severity and functional limitations, many of which are expressed through idiomatic language or tied to culturally specific daily activities (e.g., cooking, dressing, or writing).

### Strenghts

The BTCS-Q was successfully translated, adapted, and validated into Romanian, and linguistic and conceptual equivalence was ensured. The sample size was more than double the customary necessary size. The test was reliable, and there was high internal consistency. Convergent and divergent validity were strongly supported by significant correlations with objective EDX measures. The use of objective measures like EDX surpasses in methodological quality studies that rely solely on subjective questionnaires for validation. At the same time, by adhering to rigorous frameworks such as the Beaton/AAOS model, international adaptations of the BCTQ (e.g., Arabic, Korean, Portuguese, Turkish, Greek, and others), the results demonstrated improved validity, stronger reliability, and enhanced patient comprehension.

This ensures that the Romanian adaptation, like its international counterparts, remains a robust tool for both clinical decision-making and cross-cultural research, while preserving comparability with the original English version.

## 5. Conclusions

The BTCS-Q version in Romanian was successfully translated, adapted, and validated, and was found to be of high reliability, high internal consistency, and high validity on a large sample of participants. It was significantly correlated with objective EDX tests and had high test–retest reliability. Therefore, the instrument proves to be a reliable measure of CTS symptom severity in clinical and research populations.

## Figures and Tables

**Table 1:** Patients’ characteristics.

Characteristic	N = 190
Age (years), mean (SD) [range]	54.47 (14.79) [19–88]
Female, *n* (%)	139 (73.16)
Body mass index (kg/m^2^), median (IQR)	27.9 (24.83–31.3),
Handedness, *n* (%)	
Right	180 (94.74)
Left	8 (4.21)
Ambidextrous	2 (1.05)
Urban, *n* (%)	142 (74.74)
Education, *n* (%)	
Primary school	32 (16.84)
High school	88 (46.32)
University	70 (36.84)
Active repetitive movements, *n* (%)	85 (44.74)
Symptom duration (months), median (IQR)	
Right	6 (0–24)
Left	3 (0–18)

SD, standard deviation; IQR, interquartile range.

**Table 2:** Results of the Boston Carpal Tunnel Questionnaire (BCTQ) severity symptom scale (SSS) and functional symptom scale (FSS) item description, item-total correlation analysis, factor loadings and uniqueness.

Item	Mean	Standard Deviation	Cronbach’s Alpha Without Each Item	Corrected Item-Total Correlations	Factor Loadings	Uniqueness
**SSS**						
1	1.7	1.11	0.95	0.87	0.87	0.24
2	1.7	1.08	0.95	0.81	0.80	0.36
3	1.5	0.90	0.95	0.84	0.83	0.31
4	1.6	0.99	0.95	0.83	0.81	0.34
5	1.6	1.01	0.96	0.79	0.76	0.42
6	2.1	1.16	0.95	0.86	0.85	0.28
7	1.8	1.00	0.95	0.80	0.80	0.36
8	2.0	1.11	0.95	0.87	0.87	0.25
9	2.1	1.21	0.95	0.88	0.88	0.23
10	1.9	1.23	0.95	0.83	0.83	0.31
11	1.6	0.96	0.96	0.73	0.73	0.46
**FSS**						
1	1.4	0.73	0.95	0.84	0.84	0.30
2	1.5	0.85	0.95	0.86	0.86	0.25
3	1.4	0.82	0.95	0.88	0.88	0.23
4	1.5	0.92	0.95	0.89	0.88	0.22
5	1.7	1.15	0.96	0.88	0.88	0.22
6	1.5	0.85	0.95	0.91	0.91	0.17
7	1.3	0.71	0.95	0.86	0.85	0.27
8	1.4	0.72	0.95	0.88	0.88	0.23

SSS, severity symptom scale; FSS, functional symptom scale.

**Table 3:** Spearman correlation coefficients between severity symptom scale (SSS) and functional symptom scale (FSS) and electrodiagnostic measurements.

	SSS	FSS	Antidromic Median NCS (DII) (ms)	Median CMAP Latency (ms)	Ulnar CMAP Latency (ms)
SSS	-	0.82 (<0.001)	−0.64 (<0.001)	0.64 (<0.001)	−0.05 (0.357)
FSS	0.82 (<0.001)	-	−0.53 (<0.001)	0.48 (<0.001)	−0.09 (0.283)
Antidromic Median NCS (DII) (ms)	−0.64 (<0.001)	−0.53 (<0.001)	-	−0.75 (<0.001)	−0.08 (0.283)
Median CMAP Latency (ms)	0.64 (<0.001)	0.48 (<0.001)	−0.75 (<0.001)	-	0.12 (0.082)
Ulnar CMAP Latency (ms)	−0.05 (0.357)	−0.09 (0.283)	−0.08 (0.283)	0.12 (0.082)	-

NCS, nerve conductivity study; CMAP, compound muscle action potential; in brackets, adjusted *p*-values by the Holm method are shown.

**Table 4:** The severity symptom scale (SSS) and functional symptom scale (FSS) of the Boston Carpal Tunnel Questionnaire observed values at the measurement time and at the retest time.

	Test	Retest	*p*-Value	ICC (95% CI)	*p*-Value
SSS, median (IQR)	1.82 (1.36–2.52)	1.86 (1.36–2.45)	0.925	0.993 (0.986–0.997)	<0.001
FSS, median (IQR)	1 (1–1.22)	1 (1–1.22)	0.160	0.998 (0.997–0.999)	<0.001

IQR, interquartile range; ICC, interclass correlation coefficient; CI, confidence interval.

## Data Availability

The data presented in this study are available upon request from the corresponding author due to privacy or ethical reasons.

## Supplementary Materials

The following supporting information can be downloaded at https://www.mdpi.com/article/10.3390/diagnostics15233036/s1. Table S1. Translation and Cultural Adaptation of the Boston Carpal Tunnel Questionnaire. Table S2. Review of Study Designs and Methodological Approaches in BCTQ Cross-Cultural Adaptation and Validation. Refs. [15,20,21,27,30,31,32,34,35,36,37,38,39,40,41,42,43,44,45,46,47,48,51,52] are cited in the supplementary materials.

## Abbreviations

The following abbreviations are used in this manuscript:

CTS	Carpal Tunnel Syndrome
NCS	Nerve conduction studies
BCTQ	Boston Carpal Tunnel Syndrome Questionnaire
CMAP	Median Compound Muscle Action Potential
SNAP	Sensory Nerve Action Potential
EFA	Exploratory factor analysis
KMO	Kaiser-Meyer-Olkin
BMI	Body mass index
SD	Standard deviation
IQR	Interquartile range

## Appendix A. Chestionarul Boston Pentru Sindromul de Tunel Carpian—Versiunea Română

### Appendix A.1. Scala de Severitate a Simptomelor (11 Întrebări)

**Partea 1/2: Scala de Severitate a Simptomelor (11 întrebări) ***	**1**	**2**	**3**	**4**	**5**
1. Cât de severă este durerea de la nivelul încheieturii/mâinii în timpul nopții?	Normal	Ușoară	Medie	Severă	Foarte severă
2. Cât de des v-a trezit din somn durerea de la nivelul încheieturii/mâinii în ultimele 2 săptămâni?	Niciodată	O dată	2–3 ori	4–5 ori	>5 ori
3. Aveți de obicei durere la nivelul încheieturii/mâinii în timpul zilei?	Normal	Ușoară	Medie	Severă	Foarte severă
4. Cât de des aveți durere la nivelul încheieturii/mâinii în timpul zilei?	Niciodată	1–2 ori/zi	3–5 ori/zi	>5 ori/zi	Durere continuă
5. Cât durează în medie un episod de durere din timpul zilei?	Normal	<10 min	10–60 min	>60 min	Durere continuă
6. Aveți senzația de amorțeală la nivelul mâinii?	Normal	Ușoară	Medie	Severă	Foarte severă
7. Aveți senzația de slăbiciune la nivelul încheieturii/mâinii?	Normal	Ușoară	Medie	Severă	Foarte severă
8. Aveți senzația de furnicături la nivelul mâinii?	Normal	Ușoară	Medie	Severă	Foarte severă
9. Cât de severă este senzația de amorțeală/furnicăturile din timpul nopții?	Normal	Ușoară	Medie	Severă	Foarte severă
10. Cât de des v-au trezit din somn senzația de amorțeală/furnicăturile în ultimele 2 săptămâni?	Normal	O dată	2–3 ori	<5 ori	>5 ori
11. Aveți dificultăți în apucarea și folosirea obiectelor mici, precum cheile sau pixul?	Fară dificultate	Dificultate ușoară	Dificultate moderată	Foarte dificil	Foarte dificil
*** Scorul de Severitate a Simptomelor** se obține prin însumarea valorilor celor 11 întrebări și împărțirea rezultatului la 11. Un scor mai mare reflectă o severitate mai pronunțată a simptomelor.					

### Appendix A.2. Scala Statusului Funcțional (8 Intrebări)

**Partea 2/2: Scala Statusului Funcțional (8 intrebări) ****	**Nicio dificultate** **(1)**	**Dificultate ușoară** **(2)**	**Dificultate moderată** **(3)**	**Dificultate severă** **(4)**	**Activitate imposibil de efectuat din cauza simptomelor** **(5)**
1. Scris	1	2	3	4	5
2. Încheierea nasturilor	1	2	3	4	5
3. Ținerea cărții în timpul cititului	1	2	3	4	5
4. Ținerea telefonului în mână	1	2	3	4	5
5. Deschiderea borcanelor	1	2	3	4	5
6. Activități casnice	1	2	3	4	5
7. Manipularea coșului cu cumpărături	1	2	3	4	5
8. Spălatul și îmbrăcatul	1	2	3	4	5
** Pentru a calcula **Scorul Statusului Funcțional**, se însumează valorile tuturor celor 8 întrebări din această secțiune, iar rezultatul se împarte la 8. Un scor mai mare indică un grad mai sever de afectare funcțională.					
