# Supplementary material for: Translation, Cross-Cultural Adaptation and Psychometric Validation of the Romanian Version of the Boston Carpal Tunnel Questionnaire
Source: Diagnostics (Basel). 2025 Nov 28;15(23):3036. doi: 10.3390/diagnostics15233036 (PMC12691194; doi:10.3390/diagnostics15233036)
Supplement: Supplementary file 1 [file diagnostics-15-03036-s001.zip › diagnostics-3998083-supplementary.pdf]

Supplementary Table S1. Translation and Cultural Adaptation of the Boston Carpal Tunnel Questionnaire

Forward Translation.	The questionnaire was independently translated from English into Romanian by three bilingual translators, each working separately. This redundancy helps identify ambiguous terms and reduces individual bias (1,2).
Review and Synthesis.	The second translator reviewed the first version, correcting inconsistencies, while the third translator further refined the synthesis. This iterative process aligns with recommendations to achieve semantic and conceptual equivalence (3).
Linguistic Quality Check.	A Romanian language teacher reviewed the draft for grammar, syntax, and punctuation. Such linguistic checks are critical to ensure clarity and readability, particularly for patients with varied educational levels (4).
Back Translation.	The Romanian version was reverse-translated into English by a native English speaker unfamiliar with the questionnaire. Back translation allows comparison with the original tool to detect mistranslations or conceptual deviations (2,5).
Expert Committee Review and Validation.	An expert panel composed of neurologists, statisticians, and methodologists carefully examined discrepancies between the back-translated and original English versions. This stage was critical to ensure that medical terminology retained its precision and that the functional items captured patients'lived experiences accurately. The involvement of a multidisciplinary team also enhanced the content validity of the Romanian version, as recommended in previous cultural adaptation studies (1,6). By incorporating feedback from both clinical and methodological experts, the process strengthened the internal consistency and construct validity of the instrument.
Final Validation and Reliability Assurance.	Following the expert review and subsequent corrections, the Romanian BCTQ underwent final validation. The approved version included the two established subscales - the Symptom Severity Scale (SSS) and the Functional Status Scale (FSS). This rigorous multi-step process ensured that the Romanian adaptation not only mirrored the conceptual structure of the original instrument but also demonstrated improved reliability and cultural appropriateness.<br>The final validated questionnaire is available in **Appendix A.**

Supplementary Table S2. Review of Study Designs and Methodological Approaches in BCTQ Cross-Cultural Adaptation and Validation

Study	Design (sample size, methodology, duration)	Strength of findings (statistical significance, generalizability)	Weaknesses or limitations
Alanazy et al. (2019), Arabic version (4)	Translation & validation; ~103 CTS patients; cross-sectional; test–retest.	High internal consistency (α>0.8); strong validity with DASH; ICC >0.85.	Single-center; limited to Saudi population; no healthy controls.
Bîrsanu et al. (2025), Romanian BCTQ (7)	Translation, cultural adaptation, validation; 31 CTS patients in Iași; prospective study with psychometric testing (Cronbach's α, KMO, Bartlett's test).	Acceptable-to-excellent reliability (α = 0.79; standardized α = 0.984); good construct validity (KMO = 0.732; Bartlett's χ^2 = 641.4, p < 0.001); items showed strong intercorrelations; adapted to Romanian socio-cultural context.	Very small sample (n=31); all patients from one region and mostly women (90% female); no healthy controls; underpowered for factor analysis; limited external validity and generalizability.
Bougea et al. (2018), Greek version (8)	Cross-sectional; 90 CTS patients; test–retest 1 week; validity vs Canterbury scale.	α=0.89–0.93; ICC=0.75–0.79; good construct validity; ROC analysis significant.	No healthy controls; Athens-based sample; modest sample size.
Bulatović et al. (2022), Serbian version (9)	Cross-sectional; 100 CTS patients; test–retest 7 days; Rasch analysis.	Excellent reliability (α>0.9); ICC>0.9; Rasch confirmed unidimensionality.	Single-country; no controls; limited external generalizability.
Campos et al. (2003), Portuguese version (6)	Validation at UNIFESP; patients pre/post surgery; correlations with grip strength, EMG.	Reproducibility and validity confirmed; responsive to surgery; consistent with original.	Sample size not clearly reported; limited generalizability.
De Kleermaeker et al. (2019), Dutch version (10)	180 CTS patients; pre-op and 6–8 months post-op; factor analysis; responsiveness.	High reliability (α=0.72–0.83); responsive (effect sizes up to 1.96); strong construct validity.	SSS multidimensionality changes post-op; weak link to grip strength.
Gökşenoğlu et al. (2018), Duruöz Hand Index (11)	55 CTS patients; validation of DHI; test–retest 1 week.	Excellent reliability (α=0.97); ICC=0.88; validity with BCTQ r=0.64.	Small sample; mostly female housewives; single-center Turkey.
Hamzeh & Alworikat (2019), Standard Arabic version (2)	101 CTS patients; cross-cultural adaptation; test–retest 2 weeks.	α=0.877–0.895; ICC=0.899–0.944; validity with DASH confirmed.	Jordan-only; no controls; excluded comorbidities.
Karabinov et al. (2020),	64 CTS patients; compared with DASH;	α=0.87–0.88; reproducibility r=0.99–	Modest sample; small surgical

Bulgarian version (12)	subgroup of 26 pre/post surgery.	1.0; validity with DASH.	subgroup; Bulgarian-only.
Kim & Lim (2015), Korean version (13)	53 CTS surgical patients; pre-op vs 3 months post-op; validity vs K-DASH.	α>0.8; responsiveness confirmed; validity with K-DASH strong.	Small sample; surgical only; single center.
Levine et al. (1993), Original BCTQ (14)	Cross-sectional development study; 100 CTS patients; psychometric validation.	Established core instrument; α>0.9; strong reproducibility; validated widely.	Initial validation limited to US sample; generalizability improved later.
Lue et al. (2014), Chinese version (15)	99 CTS patients; 51 for reliability, 23 for responsiveness; compared with DASH, SF-36.	ICC=0.81–0.83; correlations with DASH ρ=0.63–0.75; responsiveness SRM=0.62–1.03.	Convenience sample; small surgical subgroup; single-country.
Matsuo et al. (2016), Brazilian Portuguese 6-item scale (16)	43 CTS patients; adaptation of CTS-6 and PPS; comprehension testing.	Improved comprehension; cultural adaptation successful.	Small pilot; limited psychometric validation; no reliability stats.
Metro North HHS (2016) – Functional Status Scale (17)	Clinical adaptation for monitoring in Brisbane hospitals.	Practical standardized form for clinical use.	Not independently validated; no psychometric testing.
Metro North HHS (2016) – Symptom Severity Scale (18)	Clinical adaptation for symptom monitoring.	Provides standardized monitoring tool.	No validation reported; not a research study.
Multanen et al. (2019), Finnish version (19)	193 surgically treated CTS patients; test–retest 2 weeks; validity with CTS-6, EQ-5D.	α=0.93; ICC>0.8; strong validity; large sample.	Floor effect in FSS; surgical patients only.
Multanen et al. (2020), Rasch analysis (20)	Rasch analysis; 193 CTS surgical patients; structural validity testing.	Confirmed dimensionality of BCTQ and CTS-6; strong statistical modeling.	Focused on surgical cases; not generalizable to untreated CTS.
Oteo-Álvaro et al. (2016), Spanish version (21)	90 CTS patients; pilot 20; retest 21; follow-up 40; validity vs EMG, dynamometer.	α=0.87–0.91; ICC=0.94–0.99; construct validity; effect sizes –1.9 to –3.3.	Ceiling/floor effects for 3 items; Spanish only; modest sample.
Park et al. (2013), Korean adaptation post-injection (5)	54 CTS patients; pre/post corticosteroid injection; test–retest 2 weeks.	α=0.915; ICC=0.844–0.931; validity with K-DASH, EQ-5D; responsiveness shown.	Small sample; short follow-up; single-center.
Salbaş & Solum (2023), Turkish 6-item version (22)	60 CTS patients; translation/adaptation; test–retest 7–10 days; compared with BCTQ, MHQ.	α=0.829; r=0.869; factor loadings 0.655–0.790; Bartlett p<0.001.	Small sample; KMO=0.629 near threshold; no comorbidities; Turkish-only.
Sezgin et al. (2006), Turkish version (1)	67 CTS patients; translation/validation; test–retest 7 days;	α=0.82–0.88; reproducibility r=0.60–0.77; construct validity confirmed.	Small sample; short retest interval; single center.

	compared with SF-36, grip strength.		
Trybus et al. (2019), Polish version (23)	218 CTS patients; 189 retested after 14 days; compared with DASH, MHQ, NCS.	α=0.91–0.92; ICC=0.85–0.87; r>0.7 with DASH/MHQ; large sample; no floor/ceiling effects.	Weak correlation with NCS (r=–0.16); ~9% dropout; Poland-only.
Ulbrichtová et al. (2019), Slovakian version (24)	32 factory workers at risk of CTS; translation/validation; compared with SF-36; retest 12 days.	α=0.86 (SSS), 0.94 (FSS); validity with SF-36; reproducibility high.	Very small sample; occupational risk group not clinical CTS; male-dominant.

References

1. Sezgin M, Incel NA, Sevim S, Çamdeviren H, As I, Erdoğan C. Assessment of symptom severity and functional status in patients with carpal tunnel syndrome: Reliability and validity of the Turkish version of the Boston questionnaire. Disabil Rehabil. **2006**;28(20):1281–6.
2. Hamzeh HH, Alworikat NA. Cross cultural adaptation, reliability and construct validity of the Boston Carpal Tunnel Questionnaire in standard Arabic language. Disabil Rehabil. **2021**;43(3):430–5.
3. Naghavi M, Wang H, Lozano R, Davis A, Liang X, Zhou M, et al. Global, regional, and national age-sex specific all-cause and cause-specific mortality for 240 causes of death, 1990-2013: A systematic analysis for the Global Burden of Disease Study 2013. Lancet. **2015**;385(9963):117–71.
4. Alanazy MH, Alaboudi M, Almaari A, Alhumayyd Z, Albulaihe H, Muayqil T. Translation and validation of the Arabic version of the boston carpal tunnel syndrome questionnaire. Neurosciences. **2019**;24(4):296–301.
5. Park DJ, Kang JH, Lee JW, Lee KE, Wen L, Kim TJ, et al. Cross-cultural adaptation of the korean version of the bostoncarpal tunnel questionnaire: Its clinical evaluation in patients with carpal tunnel syndrome following local corticosteroid Injection. J Korean Med Sci. **2013**;28(7):1095–9.
6. Campos CC de, Manzano GM, Andrade LB de, Castelo Filho A, Nóbrega JAM. Tradução e validação do questionário de avaliação de gravidade dos sintomas e do estado funcional na síndrome do túnel do carpo. Arq Neuropsiquiatr. **2003**;61(1):51–5.
7. Bîrsanu L, Covali R, Roman EM, Chirap-Mitulschi IA, Ciubotaru A, Turliuc MD. Romanian validation study of the Boston questionare in patients with carpal tunnel syndrome. Balneo PRM Res J. **2024**;15(4):1–16.
8. Bougea A, Zambelis T, Voskou P, Katsika PZ, Tzavara C, Kokotis P, et al. Reliability and validation of the greek version of the boston carpal tunnel questionnaire. Hand. **2018**;13(5):593–9.
9. Bulatovic D, Nikolic D, Hrkovic M, Filipovic T, Cirovic D, Radosavljevic N, et al. Reliability, Validity and Temporal Stability of the Serbian Version of the Boston Carpal Tunnel Questionnaire. Med. **2022**;58(11):1–11.
10. De Kleermaeker FGCM, Levels M, Verhagen WIM, Meulstee J. Validation of the Dutch Version of the Boston Carpal Tunnel Questionnaire. Front Neurol. **2019**;10:1–8.
11. Gökşenoğlu G, Paker N, Çelik B, Buğdaycı D, Demircioğlu D, Kesiktaş N. Reliability and validity of Duruoz Hand Index in carpal tunnel syndrome. Turkish J Phys Med Rehabil. **2018**;64(3):277–83.
12. Karabinov V, Slavchev SA, Georgiev GP. Translation and Validation of the Bulgarian Version of the Boston Carpal Tunnel Questionnaire. Cureus. **2020**;12(10).
13. Kim JK, Lim HM. The Korean version of the Carpal Tunnel Questionnaire. Cross cultural adaptation, reliability, validity and responsiveness. J Hand Surg Eur Vol. **2015**;40(2):200–5.
14. Levine DW, Simmons BP, Koris MJ, Daltroy LH, Hohl GG, Fossel AH, et al. A self-administered questionnaire for the assessment of severity of symptoms and functional status in carpal tunnel syndrome. J Bone Joint Surg Am. **1993**;75(11):1585–92.
15. Lue YJ, Lu YM, Lin GT, Liu YF. Validation of the Chinese version of the boston carpal tunnel questionnaire. J Occup Rehabil. **2014**;24(1):139–45.
16. Matsuo RP, Fernandes CH, Meirelles LM, Raduan Neto J, dos Santos JBG, Fallopa F. Translation and Cross-

Cultural Adaptation of the 6-Item Carpal Tunnel Syndrome Symptoms Scale and Palmar Pain Scale Questionnaire Into Brazilian Portuguese. Hand. **2016**;11(2):168–72.
17. Metro North HHS (**2016**) – Symptom Severity Scale .pdf.
18. Metro North HHS (**2016**) – Functional Status Scale .pdf.
19. Multanen J, Ylinen J, Karjalainen T, Kautiainen H, Repo JP, Häkkinen A. Reliability and Validity of The Finnish Version of The Boston Carpal Tunnel Questionnaire among Surgically Treated Carpal Tunnel Syndrome Patients. Scand J Surg. **2020**;109(4):343–50.
20. Multanen J, Ylinen J, Karjalainen T, Ikonen J, Häkkinen A, Repo JP. Structural validity of the Boston Carpal Tunnel Questionnaire and its short version, the 6-Item CTS symptoms scale: A Rasch analysis one year after surgery. BMC Musculoskelet Disord. **2020**;21(1):1–14.
21. Oteo-Álvaro Á, Marín MT, Matas JA, Vaquero J. Validación al castellano de la escala Boston Carpal Tunnel Questionnaire. Med Clin (Barc). **2016**;146(6):247–53.
22. Salbas E. Reliability and Validity of the Turkish Version of the 6-item Carpal Tunnel Syndrome Symptoms Scale. **2023**;326–31.
23. Trybus M, Koziej M, Belka M, Bednarek M, Banach M. The Polish version of the Boston Carpal Tunnel Questionnaire: Associations between patient-rated outcome measures and nerve conduction studies. J Plast Reconstr Aesthetic Surg. **2019**;72(6):924–32.
24. Ulbrichtová R, Jakušová V, Švihrová V, Dvorštiaková B, Hudečková H. Validation of the Slovakian version of Boston Carpal Tunnel Syndrome Questionnaire (BCTSQ). Acta medica Hradec Kral. **2019**;62(3):105–8.
